# Revolutionizing anemia detection: integrative machine learning models and advanced attention mechanisms

**DOI:** 10.1186/s42492-024-00169-4

**Published:** 2024-07-17

**Authors:** Muhammad Ramzan, Jinfang Sheng, Muhammad Usman Saeed, Bin Wang, Faisal Z. Duraihem

**Affiliations:** 1 School of Computer Science and Engineering, Central South University, Changsha, 410017 Hunan China; 2 Department of Mathematics, College of Science, King Saud University, Riyadh, 11451 Saudi Arabia

**Keywords:** Anemia, Noninvasive, Multimodal, Feature fusion, Attention module

## Abstract

This study addresses the critical issue of anemia detection using machine learning (ML) techniques. Although a widespread blood disorder with significant health implications, anemia often remains undetected. This necessitates timely and efficient diagnostic methods, as traditional approaches that rely on manual assessment are time-consuming and subjective. The present study explored the application of ML – particularly classification models, such as logistic regression, decision trees, random forest, support vector machines, Naïve Bayes, and k-nearest neighbors – in conjunction with innovative models incorporating attention modules and spatial attention to detect anemia. The proposed models demonstrated promising results, achieving high accuracy, precision, recall, and F1 scores for both textual and image datasets. In addition, an integrated approach that combines textual and image data was found to outperform the individual modalities. Specifically, the proposed AlexNet Multiple Spatial Attention model achieved an exceptional accuracy of 99.58%, emphasizing its potential to revolutionize automated anemia detection. The results of ablation studies confirm the significance of key components – including the blue-green-red, multiple, and spatial attentions – in enhancing model performance. Overall, this study presents a comprehensive and innovative framework for noninvasive anemia detection, contributing valuable insights to the field.

## Introduction

Anemia is a condition characterized by a decrease in red blood cells (RBCs) containing hemoglobin (Hb) in the bloodstream. According to the World Health Organization (WHO), anemia is classified by Hb levels of < 13 g/dL in men, 12 g/dL in women, and < 11 g/dL in children [1]. This condition commonly arises from either a decline in the production of RBCs, or a loss of RBCs that exceeds normal levels [2]. Anemia manifests as fatigue, weakness, and more severe complications if left undetected. The impact of anemia on human health profoundly influences cognitive functioning, cardiovascular health, and overall well-being. According to the WHO [3], achieving a comprehensive and reliable assessment of anemia on a global scale, as well as determining its full scope and impact, represents a significant challenge even in economically developed regions.

Traditional methods for detecting anemia rely on manual assessments – such as blood tests, microscopic examination, and collection of intravenous blood from a vein followed by hemogram analyses [4–6] – which are not only time-consuming, but also prone to subjective interpretation and error. Furthermore, invasive procedures are painful and difficult to coordinate when performed regularly [7]. The complexities inherent to accurately identifying the diverse causes and variations of anemia are further compounded by challenges in resource-constrained settings [8]. Although, machine learning (ML) techniques represent a promising avenue towards more efficient, accurate, and accessible detection of anemia [9–12], the integration of these technologies faces hurdles related to data quality, model generalization, and ethical considerations, necessitating a nuanced approach to harness their full potential [13, 14].

ML techniques can be employed to leverage algorithms and computational models for the analysis of vast datasets encompassing blood parameters, imaging results, and clinical information [15–17]. These models can identify intricate patterns and correlations that may escape human observation, thereby achieving the accurate and early detection of anemia. ML can also enhance diagnostic precision through techniques such as supervised learning, wherein algorithms learn from labeled data, and unsupervised learning, wherein patterns are detected without predefined labels. However, challenges persist in optimizing these models for diverse populations, ensuring data quality, and establishing ethical guidelines for deployment in healthcare settings [18, 19]. Nonetheless, the potential of ML to revolutionize anemia detection lies in its ability to augment traditional methods, thereby offering quicker, more scalable, and potentially more accurate diagnostic capabilities.

### Literature review

The literature review on noninvasive anemia detection encompasses a range of methodologies and datasets, each contributing valuable insights to the field. Noteworthy examples include the application of Naïve Bayes to self-collected images, yielding a notable accuracy of 92.3% [20]. In contrast, ref. [21]. employed the ResNet50 and ViT models on the CP-AnemiC dataset from Ghana, achieving an accuracy of 84.79% and F1 score of 0.837. The use of an artificial neural network (ANN) on a self-collected dataset in ref. [22] demonstrated a commendable accuracy of 97%. Furthermore, ref. [9] employed palpable palm image datasets from Ghana, utilizing a convolutional neural network (CNN) to achieve an impressive accuracy of 99.12% and F1 score of 99.89%. Another method, developed in ref. [23], was used to process 1,738,759 English tweets using the SMO algorithm, resulting in a high accuracy of 98.96%, with a precision of 96%. The diversity of these techniques is further represented by ref. [24], which utilized a Kaggle dataset with Naïve Bayes to achieve an accuracy of 90% with precision, recall, and F1 score values of 90.8%, 90.6%, and 90.6%, respectively. Moreover, ref. [25] employed a random forest on data from Jawaharlal Nehru Technological University, attaining an accuracy of 98%.

Recently, the use of computerized algorithms for the estimation of Hb content and detection of medical conditions has become related to high accuracy achieved by these algorithms in analyzing the colors of nail beds [26] from digital photographs taken by smartphones. By classifying the basic set of algorithms, a quintessence of classification methods was considered, including the support vector machine (SVM) [27], k-nearest neighbors (KNN) [18], Bayesian networks [10], ANNs [15], and decision tree [11] classifiers. Comprehensive data mining and a group of essentiality types indicate that the best algorithms for classification and detection are based on the domain of the problem to be solved.

## Methods

Deep learning (DL) has emerged as a transformative force within the healthcare sector, offering a multitude of promising applications including noninvasive techniques for the detection of anemia [28]. By harnessing the power of deep neural networks, healthcare professionals can access vast repositories of medical data, enabling more accurate diagnoses and timely interventions [29]. These cutting-edge technologies facilitate the analysis of diverse data sources, such as medical images and clinical records, to provide a more comprehensive understanding of patient health [30]. With a capacity to process complex multimodal data, DL is poised to revolutionize anemia detection, making it more accessible, efficient, and patient-friendly than ever before.

### Logistic regression

The logistic regression (LR) is a statistical method for binary classification that models the relationship between a binary outcome variable and predictor variables and is given in Eq. (1).

1 $$P\left(Y=1\right)\;=\;\frac1{\left(1+e^\wedge\left(-z\right)\right)}$$

It uses a logistic function to estimate the probability of an outcome being 1 (positive class) based on a linear combination of predictors [31]. LR is generally implemented on balanced datasets, with performance measures including imbalance, undersampling, oversampling, SMOTE, and ADASYN.

### Decision tree classifier

Decision trees [32] are supervised learning methods that perform classification and regression tasks by partitioning the feature space into segments based on the learned rules to generate predictions. The tree structure consists of nodes representing features, branches representing decision rules, and leaf nodes containing output values or class labels. At each node, the algorithm selects the best feature to split the data based on measures such as Gini impurity or entropy. For classification, the decision rule at each node can be represented as given in Eq. (2).

2 $$if\;x_i\leq threshold:go\;left,\;else\;go\;right\;$$

where *x*_*i*_ is a feature and the threshold is determined during training. This recursive splitting process continues until a stopping criterion is satisfied, creating a hierarchical structure that facilitates prediction by traversing the tree based on input features, ultimately reaching a leaf node with the predicted output.

### Random forest classifier

The random forest [33] is an ensemble learning method that builds multiple decision trees during training, each of which is trained on a random subset of the training data, with bagging used to prevent overfitting. To generate predictions, it averages outputs for regression or uses voting for classification, leading to enhanced accuracy and robustness and can be calculated using Eq. (3). The random forest excels in handling high-dimensional data, reducing variance, and maintaining strong generalization while being resilient to noise during training.

3 $$\widehat Y\;=\;model\;\left(f_1\left(x\right),\;f_2\left(X\right),\;\cdots\;,\;f_N\left(X\right)\right)$$

### SVM

The SVM excels in classification and regression by finding the optimal hyperplanes in high-dimensional spaces and maximizing the margin for better generalization [34]. It accommodates linear and nonlinear relationships using diverse kernel functions given in Eq. (4), thereby facilitating separation in higher dimensions. With its optimal decision boundary, the SVM is robust against overfitting and performs well in complex scenarios, making it a versatile and widely used ML tool.

4 $$f\left(x\right)=sign\;\left({\textstyle\sum_{i=1}^n}\;a_iy_iK\left(x_,x_i\right)\;+\;b\right)$$

### Naïve Bayes

Naïve Bayes [35], which is rooted in Bayes’ theorem, assumes feature independence and delivers effective classification results across diverse applications. The class probabilities are computed by Eq. (5), considering the conditional probabilities of class features. Despite its simplistic assumptions, Naïve Bayes excels in text classification and spam filtering. Owing to its efficiency, suitability for large datasets, and minimal training data requirements, this model is widely used in resource-constrained settings.

5 $$P(C_k\left|x_1\right.,x_2,\dots,x_n)=\frac{P\left(C_k\right)\cdot P\left(x_1\left|C_k\right.\right)\cdot P\left(x_2\left|C_k\right.\right)\cdot\dots\cdot P(x_n\left|C_k\right.)}{P\left(x_1\right)\cdot P\left(x_2\right)\cdot\dots\cdot P(x_n)}$$

### KNN

KNN is a simple yet powerful supervised learning method for classification and regression tasks, which predicts the labels or values for new data points based on the majority or average of their KNN [36] in the feature space using distance metrics such as the Euclidean or Manhattan distance given in Eq. (6). Despite its flexibility and ease of implementation, KNN can be computationally expensive with large datasets owing to the memory usage and distance calculations for each prediction.

6 $$\widehat{y}= mode({y}_{1},{y}_{2},\dots ,{y}_{k})$$

### Proposed model using blue-green-red

**Fig. 1:**
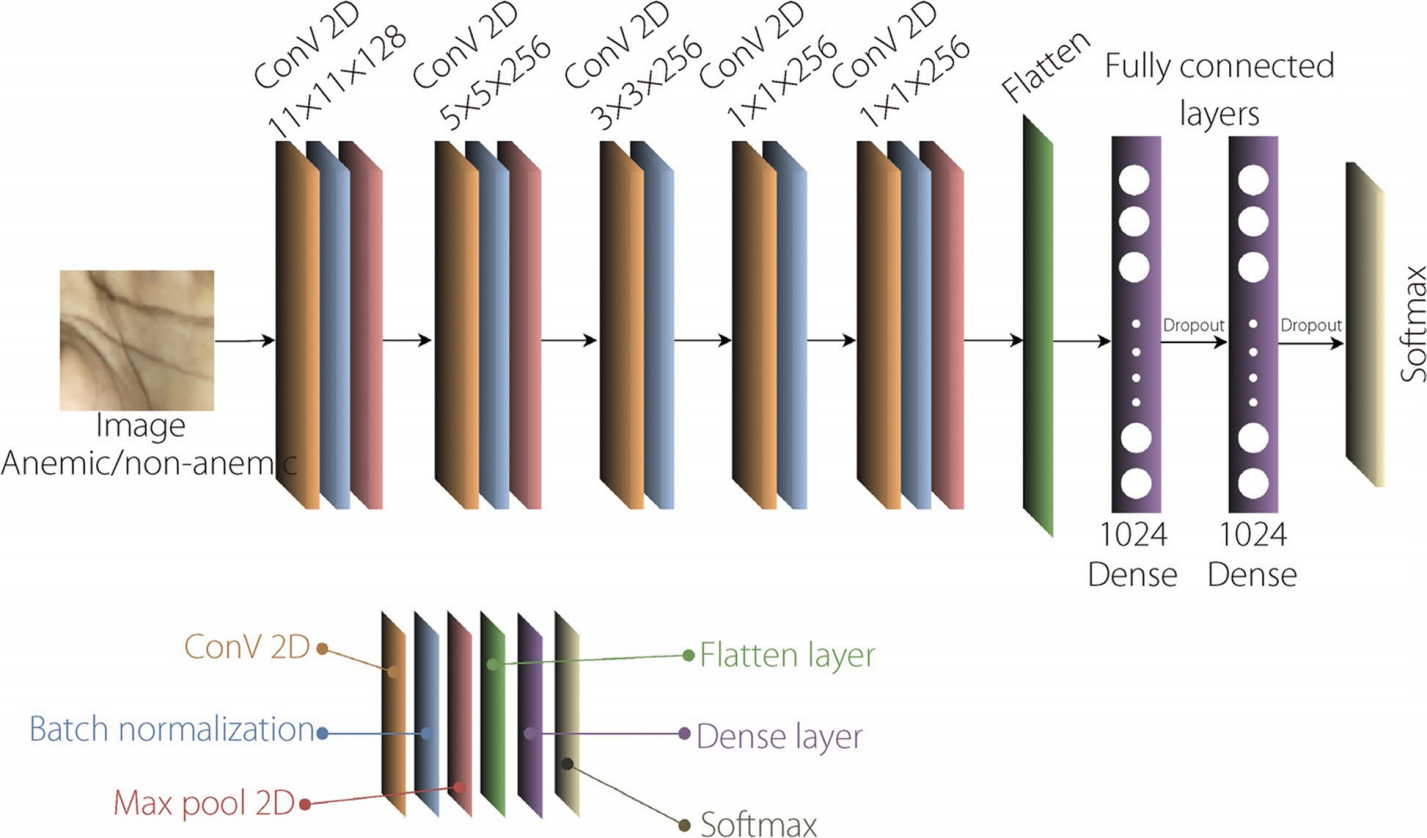
Proposed BGR network architecture for anemia detection

A modified variant of AlexNet was developed by deploying a CNN on the blue-green-red (BGR) color channels. The model features a 128-filter convolutional layer (11 × 11 kernels), rectified linear unit (ReLU) activation, and batch normalization. Further layers include convolution, batch normalization, and strategic max pooling. Fully connected layers with ReLU activation and dropout are also employed to address overfitting. The classification employs Softmax activation. The proposed model with a BGR channel is illustrated in Fig. 1.

### Proposed model using attention module

**Fig. 2:**
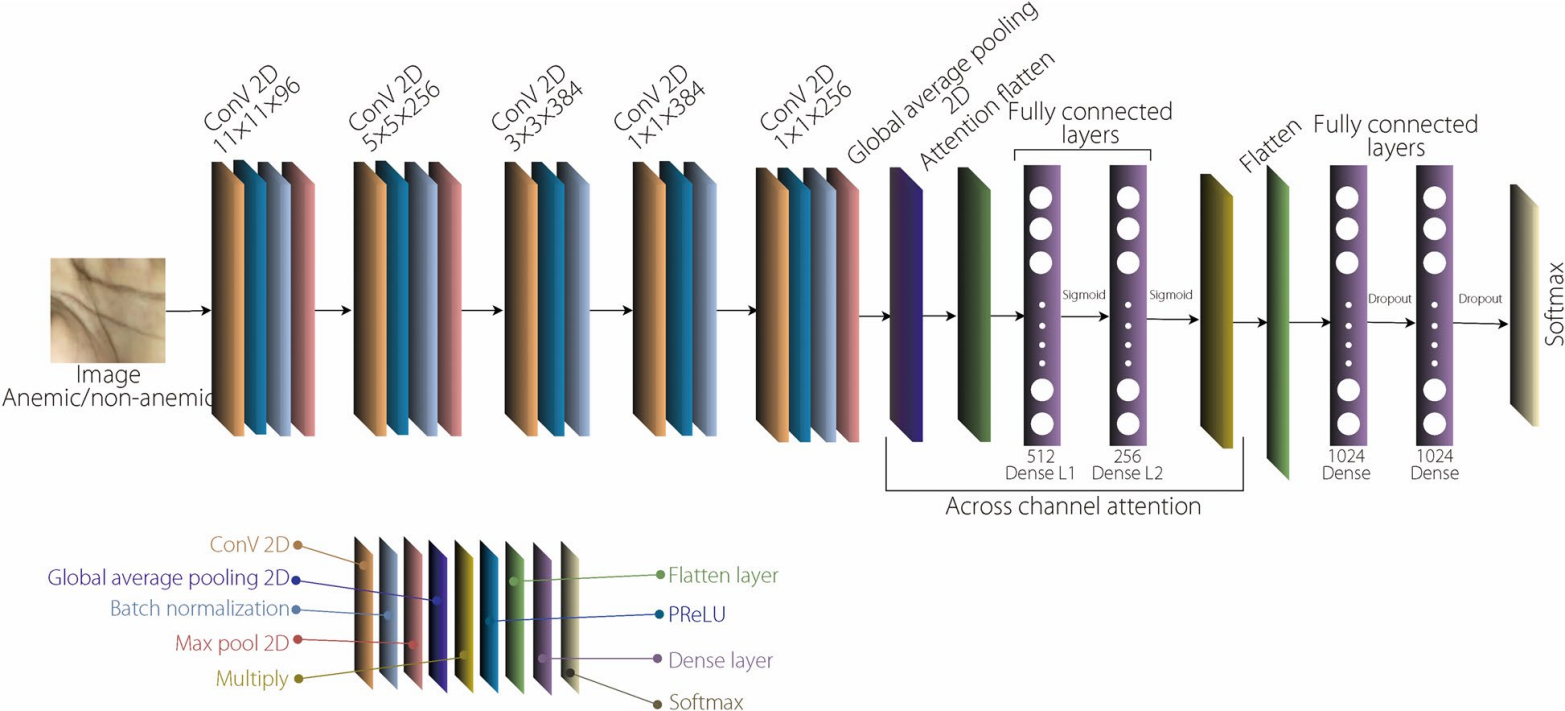
Proposed attention-based model architecture for anemia detection

This model, representing another modification of AlexNet, includes an attention module after the last pooling layer, as shown in Fig. 2. The model preprocesses input images by rescaling pixel values to [0, 1], and then proceeds through the convolutional and pooling layers. The attention module employs global average pooling to aggregate spatial information, and calculates channel-wise attention weights through fully connected layers with L1 and L2 regularization. The ‘Multiply’ operation highlights relevant features across channels. The final feature representation is classified via fully connected layers with dropout, outputting class probabilities using Softmax activation.

### Proposed model using multiple-channel attention module

**Fig. 3:**
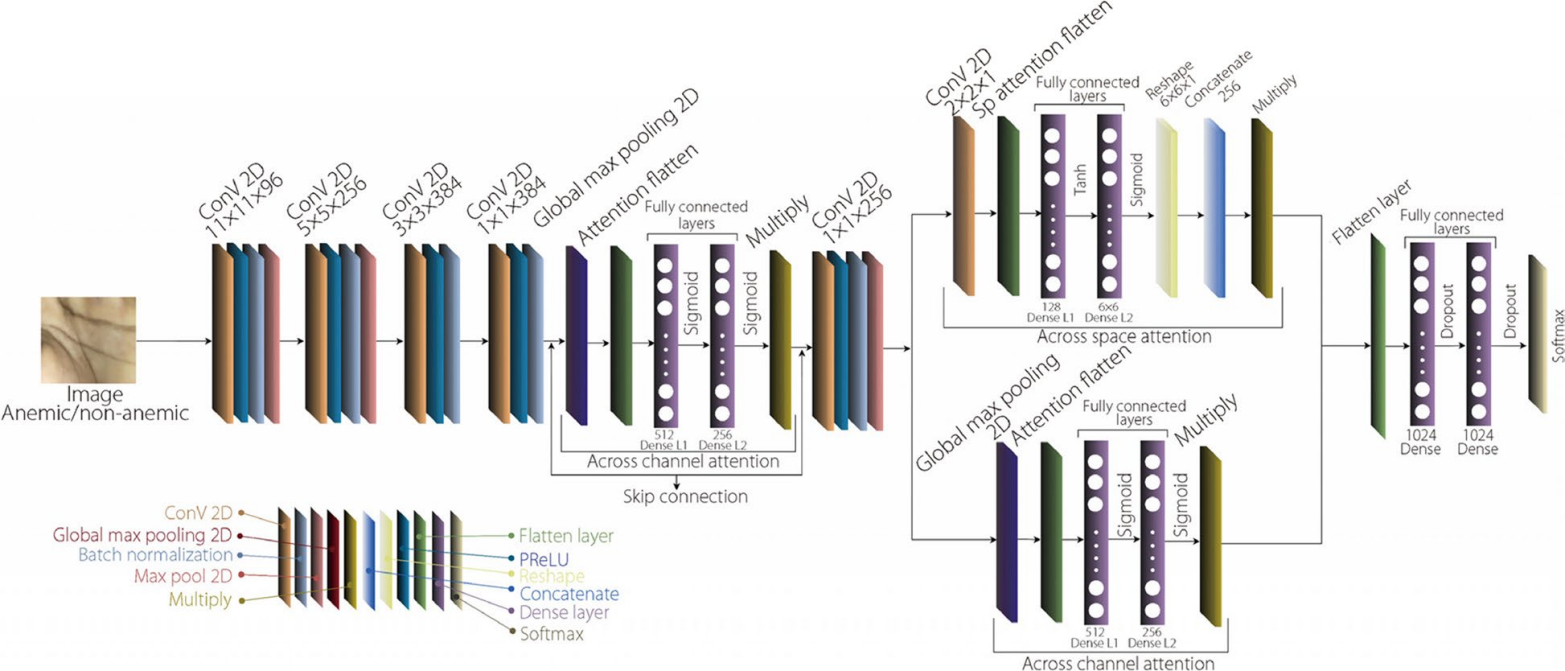
Proposed AlexNet architecture with multiple attention modules

This modification of AlexNet incorporates a multilayer channel attention module for enhanced feature selection, encompassing four convolutional layers with parametric rectified linear unit (PreLU) activation, batch normalization, and max pooling. An attention module that integrates channel-wise and spatial attention mechanisms is introduced after the fourth convolutional layer. Channel-wise attention is computed using the global max pooling and dense layers, followed by element-wise multiplication with feature maps. The model includes additional convolutional layers, PReLU activations, batch normalization, max pooling, and channel-wise attention for the refined feature maps. Three dense layers with dropout complete the classification process by combining the classic AlexNet architecture with attention modules for improved performance. The proposed model is illustrated in Fig. 3.

### Proposed model with spatial attention module

**Fig. 4:**
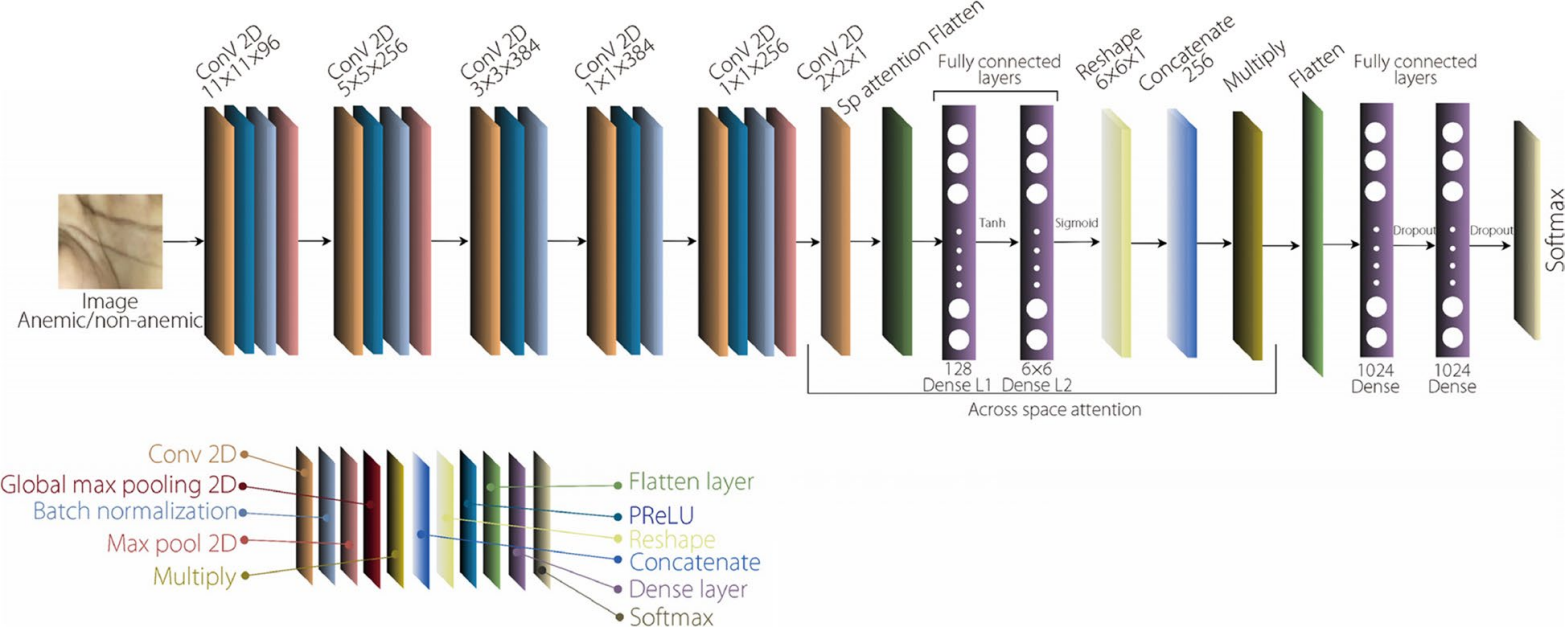
Proposed network architecture with AlexNet and spatial attention module

This model combines the AlexNet architecture with a spatial attention module for anemia detection. It begins with input layer rescaling and builds convolutional layers using PReLU activation and batch normalization for feature extraction. The max-pooling layers downsample the feature maps. The spatial attention module, which is a convolutional layer with a (2, 2) kernel size, generates a spatial attention map that passes through two dense layers with tanh and sigmoid activations, is reshaped to match the feature map dimensionality, and is concatenated 256 times for element-wise multiplication with the feature maps. This enhances relevant spatial regions by introducing spatial attention. The feature maps undergo flattening and pass through two fully connected layers (L1 and L2) with ReLU activation and dropout regularization for high-level feature capture. The final layer (L3) employs Softmax activation for multiclass classification, enhancing spatial awareness and feature localization. Figure 4 depicts the proposed architecture for anemia detection.

### Proposed AlexNet-based multiple spatial attention model for automated anemia detection

**Fig. 5:**
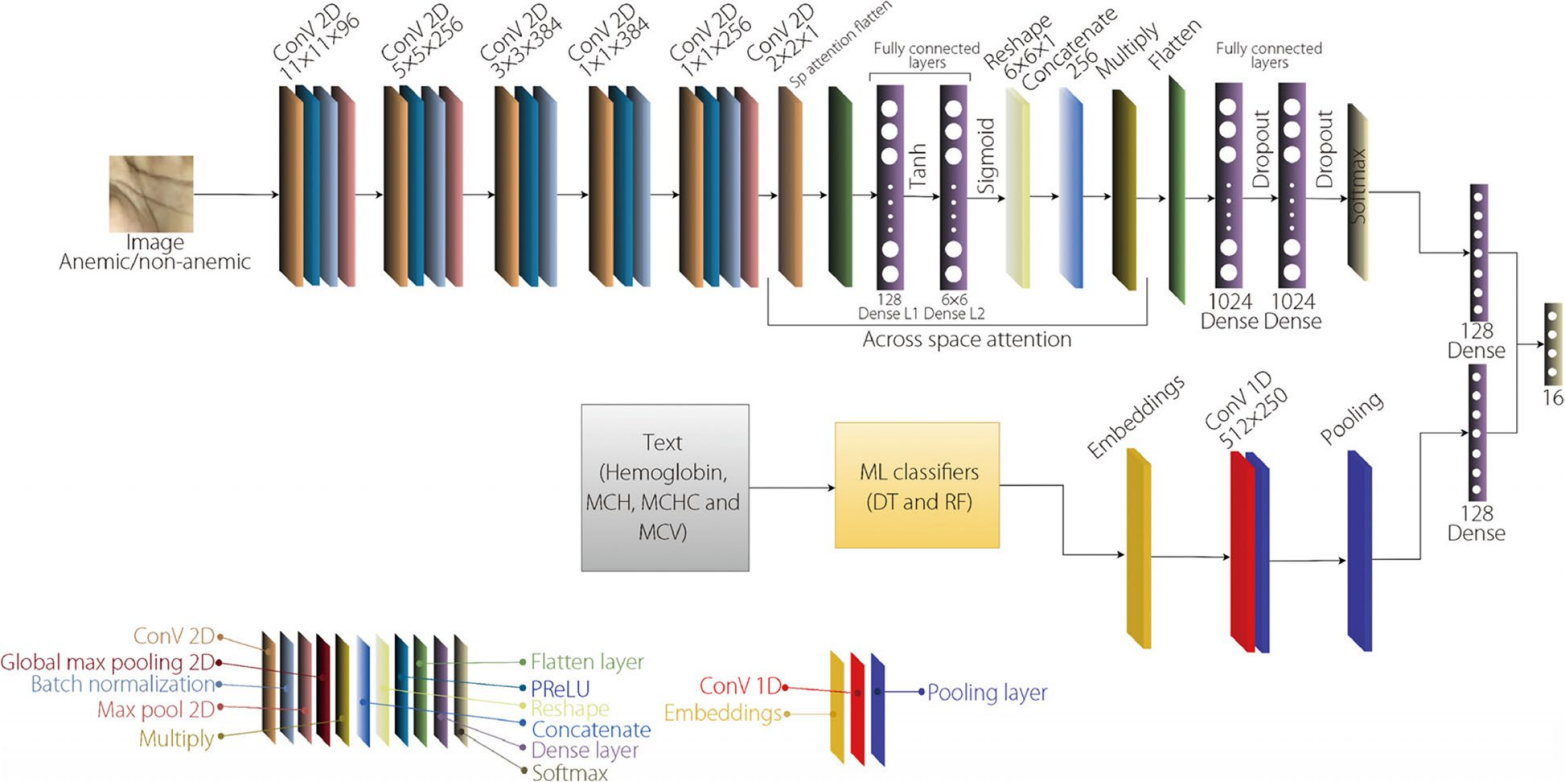
Proposed AMSA network architecture

The proposed AlexNet-based multiple spatial attention (AMSA) architecture integrates various processing techniques to classify anemia. Textual blood test data are analyzed using ML classifiers, such as decision trees and random forests, whereas image data – generally representing palm features – are processed using a CNN that extracts relevant information through a series of convolutional and pooling layers. Attention mechanisms are employed within the network to emphasize crucial image regions. Subsequently, the features extracted from both textual and image data are transformed and merged through the embedding layers. Finally, the combined information is processed through the fully connected layers, culminating in the model’s classification of the blood sample as anemic or non-anemic. Figure 5 illustrates the detailed network architecture of the proposed AMSA model.

## Results and Discussion

### Textual dataset

**Fig. 6:**
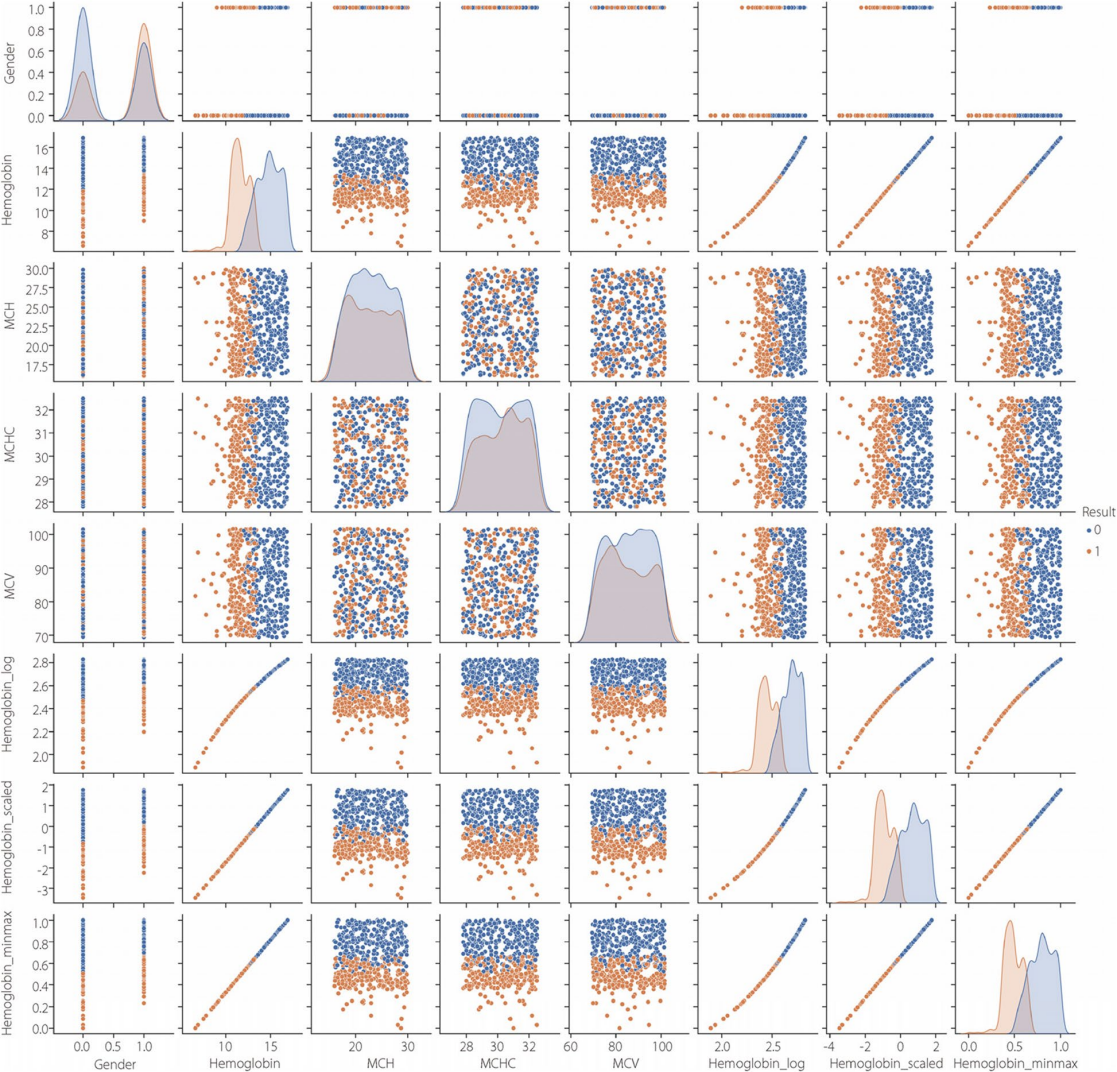
Pairplots representing relationships between variables. Hb presents a kind of constant slope with another variable for both anemic and non-anemic data

Two dataset modalities were utilized, with results combined to evaluate and classify anemia. The textual data used in this study were obtained from the Kaggle anemia dataset [37], which was curated to predict anemia susceptibility using a binary classifier. The key variables included sex (0 for male, 1 for female), Hb level, mean corpuscular hemoglobin (MCH), mean corpuscular hemoglobin concentration (MCHC), mean corpuscular volume (MCV), and results (0 for non-anemic, 1 for anemic). The dataset was tailored for ML applications, specifically geared towards forecasting anemia based on the aforementioned attributes, providing a valuable resource for medical research and diagnosis, as shown in Fig. 6.

### Image dataset

**Fig. 7:**
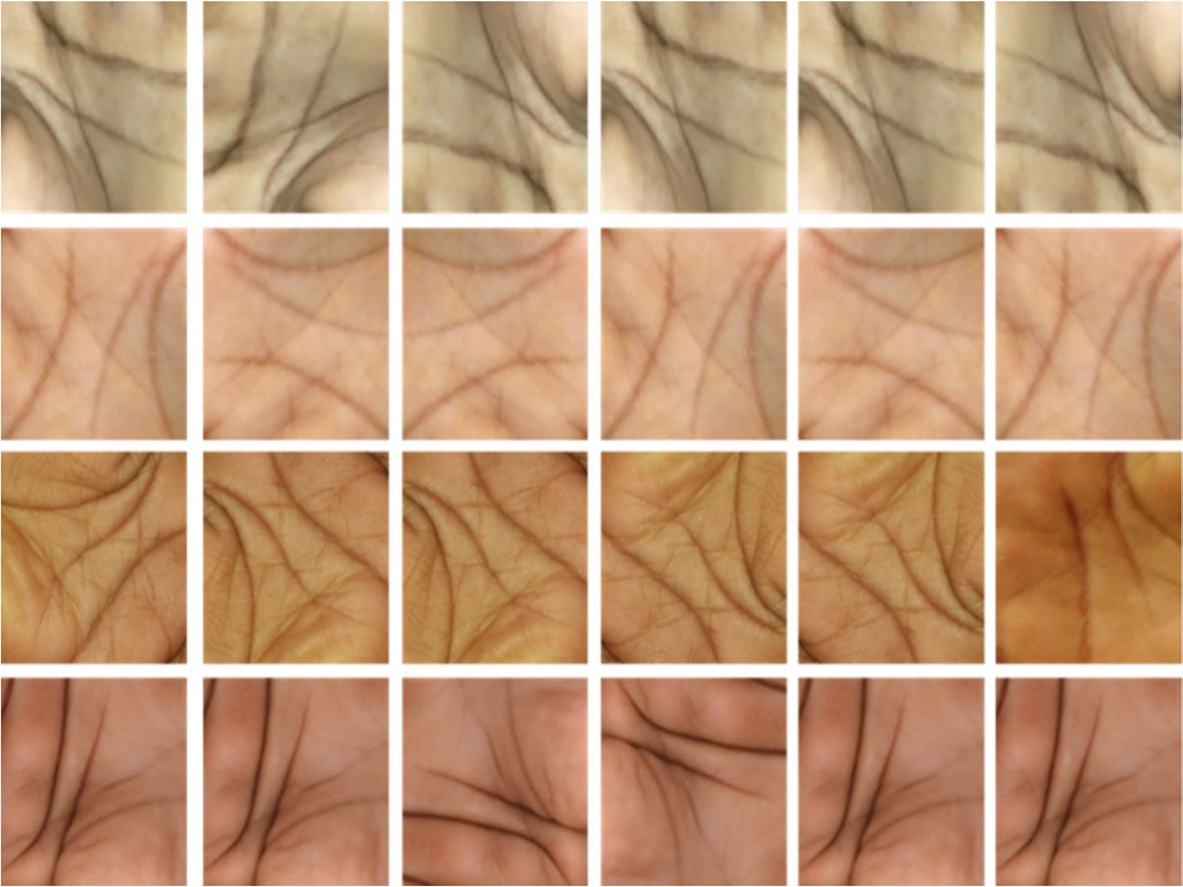
Labeled image data

The image dataset used in this study was adopted from an open-source repository available online, which has already been preprocessed for ease of integration into the investigative framework. The dataset, sourced from ref. [38] and available on Mendeley data, underwent meticulous preprocessing to minimize noise and irrelevant information while optimizing its suitability for the research objectives, as shown in Fig. 7. The decision to employ the dataset in its existing processed state was grounded in the aim of maintaining transparency and reproducibility, as raw data and preprocessing techniques are openly accessible. This approach not only facilitates the seamless replication of experiments by fellow researchers, but also underscores the commitment to methodological clarity in the pursuit of reliable and robust results.

### Evaluation metrics

#### Accuracy

Accuracy represents the proportion of correctly predicted instances (both true positives and true negatives) out of all instances, providing a general measure of how well the model performs across all classes. The mathematical equation for accuracy is

$$Accuracy=\frac{(TP+TN)}{(TP+TN+FP+FN)}$$

#### Precision

Precision measures the proportion of correctly predicted positive instances among all instances predicted to be positive, focusing on the quality of positive predictions. This measure can be calculated as

$$Precision=\frac{TP}{(TP+FP)}$$

#### Recall

Recall, also known as sensitivity or the true positive rate, measures the proportion of correctly predicted positive instances out of all actual positive instances, representing the model’s ability to identify positive instances. It is calculated as:

$$Recall=\frac{TP}{(TP+FN)}$$

#### F1 score

The F1 score is the harmonic mean of precision and recall, providing a balanced measure of the model’s performance. By accounting for both precision and recall, the F1 score is suitable when there is an imbalance between positive and negative instances.

$$F1\ score=2\frac{(Precision*Recall)}{(Precision+Recall)}$$

### Preprocessing

#### Textual data

The textual dataset, which initially encompassed 1421 instances spanning six columns, was preprocessed to prepare the data for ML. Key features such as Hb, MCH, MCHC, and MCV were scrutinized for insights into anemia, utilizing statistical tests (t-tests, odds ratio, and *χ* ^2^) to examine variable relationships. Feature selection methods – including correlation-based selection, SelectKBest, and the extra tree classifier – identified ‘Hb,’ ‘Sex,’ and ‘MCV’ as pivotal features. To enhance accuracy and interpretability, feature scaling, standardization, normalization, and logarithmic transformations were applied. Data imbalance was addressed using techniques including random oversampling, random undersampling, SMOTE, and ADASYN to improve detection performance.

#### Image data

The Mendeley dataset underwent preprocessing by extracting regions of interest using the threshold triangle method and categorizing the data as anemic or non-anemic based on the Hb results. Image augmentation expanded the dataset from 710 to 4260, each labeled with an anemic or non-anemic identifier. These steps were completed before integration to maintain the integrity of the dataset.

#### Feature fusion with textual and image data

**Fig. 8:**
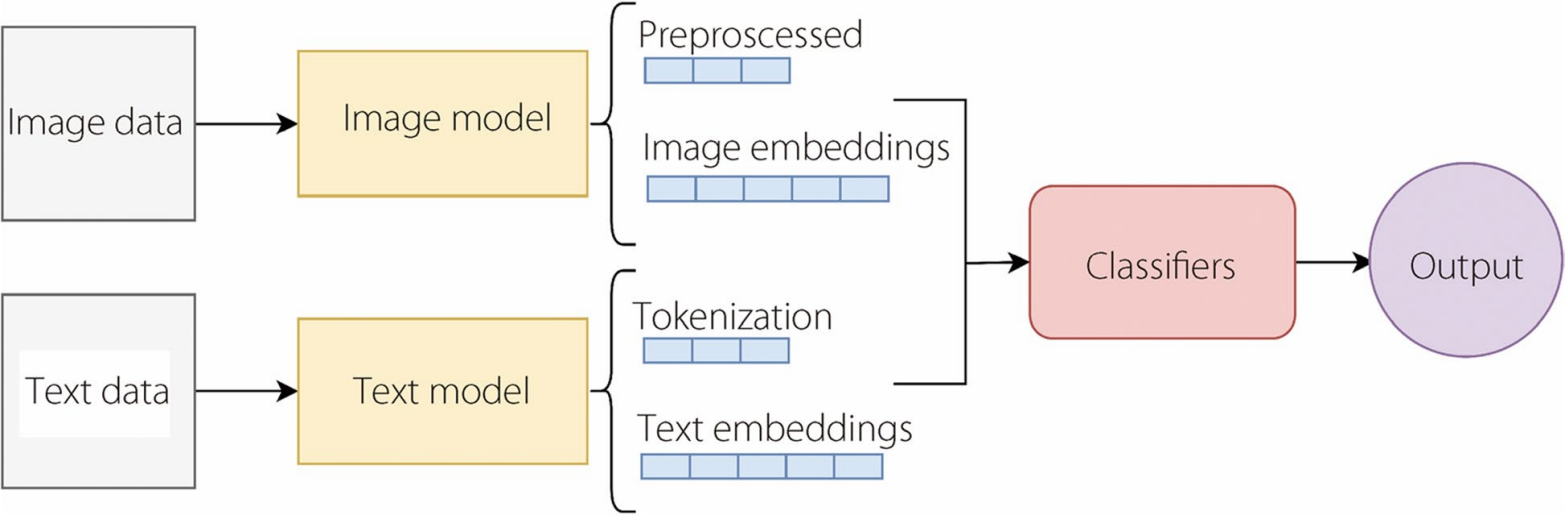
Feature fusion of textual and image data

The fusion of the textual and image data involves a systematic integration process for harnessing complementary information present in both modalities, as shown in Fig. 8. First, textual data, consisting of patient-relevant metadata, were preprocessed, tokenized, and transformed into numerical representations using word embeddings. Simultaneously, the palm image data were preprocessed using computer vision methods. The fusion of the processed textual and image data occurs at a higher representation level, where the learned embeddings and extracted image features are concatenated. This combined representation captures nuanced relationships between textual descriptions and visual patterns, enriching the understanding of the model and enabling it to generate more accurate predictions for anemia detection. Figure 8 shows the fusion of the textual and image data.

### Performance results on textual and image data

The results obtained using textual data underscore the importance of features such as Hb level, sex, and MCV in detecting anemia. Notably, the decision tree exhibited strong performance irrespective of measures used to handle imbalanced data, and the SVM also exhibited promising accuracy. Decision trees stand out for their accuracy and interpretability, offering insights into the decision-making process. The model hyperparameters were optimized using GridSearchCV to enhance performance. Furthermore, these results reveal a higher risk of anemia in females compared to that in males, as supported by odds ratio calculations. Interestingly, data scaling had a minimal impact on model performance, indicating the robustness of the algorithm to feature-scale variations. The LR, decision tree, random forest, SVM, Gaussian naïve Bayes, and KNN algorithms all provide unique insights into feature importance, aiding in the prioritization of important features for subsequent analyses and providing interpretability for classification processes.

The results obtained using image data encompass various adaptations of the AlexNet architecture, each designed to enhance feature extraction and classification accuracy. Among these, the model that integrates a spatial attention module stands out for its superior performance. By incorporating a mechanism to selectively emphasize relevant spatial regions within images, this model effectively boosts the focus of the network on crucial features for anemia detection. Unlike the channel-wise and global attention mechanisms typically employed in ML, the spatial attention module enables the network to dynamically adjust its attention based on the spatial context of each input image. This finer granularity in feature selection allows for a more precise discrimination between healthy and anemic regions within the images, leading to improved classification accuracy. In addition, the spatial attention module seamlessly complements the feature extraction capabilities of the AlexNet architecture, thereby maximizing the utilization of both spatial and channel-wise information to enhance detection performance. Consequently, the model’s ability to incorporate spatial awareness and feature localization through the spatial attention module contributes significantly to its high accuracy. The results obtained by different models for the textual and image datasets are listed in Table 1, with the best results for each type of data denoted in bold.

**Table 1:** Model performance on text and image data

Model	Dataset	Accuracy	Precision	Recall	F1 score
LR	Text	92.36%	96.19%	95.63%	95.19%
DT		**95.62**%	**98.60**%	**97.92**%	**98.01**%
RF		91.43%	94.36%	95.09%	94.98%
SVM		94.06%	97.62%	98.19%	97.67%
NB		93.16%	95.65%	94.97%	96.31%
KNN		94.67%	96.49%	96.75%	95.34%
AlexNet + BGR	Image	83.16%	86.71%	87.34%	86.48%
AlexNet + attention		82.37%	85.47%	86.13%	85.79%
AlexNet + multiple channel attention		84.62%	87.32%	86.97%	87.67%
AlexNet + spatial attention		**86.97**%	**90.71**%	**89.67**%	**88.93**%

### Performance of proposed model using feature fusion

A comprehensive approach was adopted to leverage the strengths of each model in the process of feature fusion by integrating textual and visual modalities. Initially, feature extraction was performed using AlexNet for image data by employing different attention mechanisms tailored to enhance feature representation. Similarly, a CNN architecture with a single convolutional layer was used to transform textual into embeddings, facilitating effective feature extraction. Subsequently, a decision-tree model was deployed for classification, incorporating the extracted features from both modalities. Notably, the integration of spatial attention modules yielded the best performance, as evidenced by the highest accuracy among the considered attention mechanisms. The results of the feature fusion approach for the four ML models are listed in Table 2, with the best results denoted in bold.

**Table 2:** Model performance using feature fusion

Model	Dataset	Accuracy	Precision	Recall	F1 score
AlexNet + BGR	Image + text	94.51%	96.78%	95.98%	96.35%
AlexNet + attention		93.78%	95.79%	96.04%	96.38%
AlexNet + multiple channel attention		94.72%	97.62%	97.18%	98.06%
AlexNet + spatial attention		**95.36**%	**98.47**%	**97.83**%	**98.65**%

### Performance of AMSA

The proposed model combines AlexNet with multiple attention modules, including spatial attention. Because the implementation of spatial attention mechanisms was previously observed to yield the highest accuracy, the subsequent investigation aimed to explore alternative attention mechanisms within different models. The objective was to enhance classification performance by integrating double-attention mechanisms into a unified model architecture by utilizing text embeddings extracted via a single convolutional layer. Ultimately, the highest accuracy was achieved by AlexNet when augmented with multiple-channel and spatial attention mechanisms, in conjunction with a single-layer CNN for text processing. This comprehensive framework underscores the efficacy of leveraging attention mechanisms in tandem with advanced neural network architectures to optimize classification accuracy. The proposed model achieved accuracy, precision, recall, and F1 score of 99.58%, 99.97%, 99.95%, and 99.97%, respectively, outperforming all other models in the detection of anemia.

### Ablation study

Ablation experiments were conducted to determine the impact of each component of the proposed model on overall performance, with quantitative results presented in Table 3. Multiple experiments were conducted, with the results considered significant.

**Table 3:** Results of ablation study with proposed AMSA model

Method	No BGR	No attention	No multiple attention	No spatial attention	Accuracy	Precision	Recall	F1 score
AMSA	√	√	√	√	**99.58%**	**99.97%**	**99.95%**	**99.97%**
No BGR	×	√	√	√	99.15%	99.85%	99. 82%	99.83%
No attention	√	×	√	√	92.64%	95.12%	95.24%	96.01%
No multiple attention	√	√	×	√	93.75%	96.37%	96.15%	95.97%
No spatial attention	√	√	√	×	90.95%	94.72%	94.51%	94.42%

#### No BGR

First, the proposed model was revised by eliminating the BGR component. Despite this modification, the model demonstrated exceptional performance, achieving high accuracy, precision, recall, and F1 score. However, the inclusion of BGR aids in extracting superior features, consequently enhancing the detection of anemia. Hence, when incorporating BGR, the proposed model not only generates more significant features but also yields improved detection results.

#### No attention

With the omission of attention, the proposed model still attained commendable performance. However, attention plays a critical role in enhancing feature extraction, leading to improved detection performance. Therefore, reinstating attention not only facilitates the generation of more significant features, but also contributes to achieving better overall detection results.

#### No multiple attention

After removing the multiple attention mechanism from the proposed model, the model still achieved notable performance metrics. However, this mechanism significantly enhances feature extraction, augmenting the effectiveness of anemia detection. Hence, integrating this mechanism into the proposed model enhances performance by facilitating the extraction of more relevant features.

#### No spatial attention

Removing spatial attention from the proposed model resulted decreasing the results of the proposed model. Because spatial attention enhances feature extraction, it is vital for generating more relevant features and better overall detection results.

### Comparison between proposed and existing methods

The comprehensive evaluation of various anemia detection models reveals a diverse array of methodologies and corresponding performance results. The AMSA model was rigorously tested using real-time data gathered during a clinical trial conducted at a hospital in Pakistan (Table 4). The trial was overseen by a healthcare professional – a doctor with three years of field experience and an MBBS degree from Shandong First Medical University, China – who also supervised the authentication and verification of experimental results.

**Table 4:** Performance of proposed model on real-time data in a clinical trial

Serial No.	Image_detail	Time	Date	Gender	Hb	MCV	Anemia condition	Result
1	IMG20240428245533	11:00	04/28/2024	0	11.2	75.2	1	Anemic
2	IMG20240428232514	11:38	04/28/2024	0	10.7	86.4	1	Anemic
3	IMG20240428282744	15:13	04/28/2024	1	11.8	78.7	1	Anemic
4	IMG20240428655511	15:15	04/28/2024	1	14.3	90.1	0	Non-anemic
5	IMG20240428282617	15:17	04/28/2024	0	13.4	95.9	0	Non-anemic

As shown in Table 5, the proposed model outperforms from state-of-the-art methods. Throughout the experiments, some significant limitations and challenges inherent to the field of ML in healthcare were encountered. The foremost challenge was the acquisition of high-quality and sufficiently large datasets to train the models. Despite leveraging datasets from sources such as Kaggle and Mendeley, constraints were present in relation to the dataset size and representativeness, which are essential for robustness. Furthermore, data imbalance poses a significant hurdle, requiring the implementation of various techniques such as oversampling, undersampling, and synthetic data generation to mitigate bias. Ensuring that the proposed models are well-generalizable to new data also proved to be a complex task, necessitating meticulous cross-validation and hyperparameter tuning. The interpretability of the proposed models also emerged as a challenge given the inherent complexity of DL architectures and the need for transparent decision making in healthcare applications. Moreover, patient privacy, data protection, and biases in algorithms are crucial ethical considerations which require careful attention throughout the research process. Finally, the integration of ML-based diagnostic tools into existing clinical workflows presents logistical and regulatory challenges that require close collaboration between researchers and healthcare professionals.

**Table 5:** Comparison of results between proposed and existing models

Ref.	Dataset	Number of data	Algorithm	Accuracy	F1 score	Precision	Recall
[20]	Self-collected	40 respondents images	Naïve Bayes	92.30%	-	-	-
[21]	CP-AnemiC (A Conjunctival Pallor) Dataset from Ghana	710 images (424 are labelled anemic and 286 as non-anemic)	ResNet50 and ViT	84.79%	83.70%	-	-
[22]	Self-collected data	3103 images (1678 non-anemic and 1425 anemic)	ANN	97.00%	-	-	-
[9]	Palpable Palm Image Datasets from Ghana	2635 images (70% training, 10% validation and 20% test data)	CNN	99.12%	99.89%	-	-
[23]	Self-collected and processed data	1,738,759 English tweets	SMO	98.96%	-	96.00%	89.00%
[24]	Kaggle Data set	500 (instances, 66% train and 34% test data)	Naïve Bayes	90.00%	90.60%	90.80%	90.60%
[25]	Jawaharlal Nehru Technological University, Hyderabad	1387 records (80% train data and 20% test data)	Random forest	98.00%	-	-	-
	Palpable Palm Image Datasets from Ghana + Kaggle Data set	4260 images data + 1421-instance (80% training data and 20% test data)	Purposed CNN model	99.58%	99.97%	99.95%	99.97%

## Conclusions

In conclusion, this study pioneers an advanced and effective approach to anemia detection using ML, showcasing the potential of innovative models and integrated datasets. The proposed AMSA model, which combines AlexNet with multiple spatial attention modules, achieved an unparalleled accuracy of 99.58%, surpassing that of the existing methods. Ablation studies were conducted to underscored the importance of key components in enhancing performance. This work not only advances the field of automated anemia detection, but also sets a benchmark for the integration of diverse datasets and model architectures in healthcare applications. These findings hold significant promise for improving diagnostic precision and addressing the global health challenges of anemia.

## Data Availability

Not applicable.

## Abbreviations

RBC: Red blood cell
ML: Machine learning
Hb: Hemoglobin
WHO: World Health Organization
ANN: Artificial neural network
CNN: Convolutional neural network
SVM: Support vector machine
KNN: k-nearest neighbors
DL: Deep learning
LR: Logistic regression
BGR: Blue-green-red
ReLU: Rectified linear unit
PReLU: Parametric rectified linear unit
AMSA: AlexNet-based multiple spatial attention
MCH: Mean corpuscular hemoglobin
MCHC: Mean corpuscular hemoglobin concentration
MCV: Mean corpuscular volume
